# The Mitochondrial Genome of *Ylistrum japonicum* (Bivalvia, Pectinidae) and Its Phylogenetic Analysis

**DOI:** 10.3390/ijms25168755

**Published:** 2024-08-11

**Authors:** Yida Han, Yaoyu Xie, Zhenlin Hao, Junxia Mao, Xubo Wang, Yaqing Chang, Ying Tian

**Affiliations:** Key Laboratory of Mariculture & Stock Enhancement in North China Sea, Ministry of Agriculture and Rural Affairs, Dalian Ocean University, Dalian 116023, China; hyd19990301@163.com (Y.H.); 19818925427@163.com (Y.X.); haozhenlin@dlou.edu.cn (Z.H.); maojunxia@dlou.edu.cn (J.M.); wangxubo@dlou.edu.cn (X.W.)

**Keywords:** saucer scallop, mitogenome, taxonomy

## Abstract

The *Ylistrum japonicum* is a commercially valuable scallop known for its long-distance swimming abilities. Despite its economic importance, genetic and genomic research on this species is limited. This study presents the first complete mitochondrial genome of *Y*. *japonicum*. The mitochondrial genome is 19,475 bp long and encompasses 13 protein-coding genes, three ribosomal RNA genes, and 23 transfer RNA genes. Two distinct phylogenetic analyses were used to explore the phylogenetic position of the *Y*. *japonicum* within the family Pectinidae. Based on one mitochondrial phylogenetic analysis by selecting 15 Pectinidae species and additional outgroup taxa and one single gene phylogenetic analysis by 16S rRNA, two phylogenetic trees were constructed to provide clearer insights into the evolutionary placement of *Y*. *japonicum* within the family Pectinidae. Our analysis reveals that *Ylistrum* is a basal lineage to the Pectininae clade, distinct from its previously assigned tribe, Amusiini. This study offers critical insights into the genetic makeup and evolutionary history of *Y*. *japonicum*, enhancing our knowledge of this economically vital species.

## 1. Introduction

The genus *Ylistrum*, a member of the phylum Mollusca, class Bivalvia, order Pectinoida, family Pectinidae, was established by Mynhardt et al. [1] in 2014 and is derived from the Greek word “ylistro”, meaning “glide”, reflecting the gliding habits of this group, and dividing the original genus *Amusium* into two genera, *Ylistrum* and *Amusium*. Both *Ylistrum* and *Amusium* are primarily found in the Indian–Pacific region and exhibit similar lifestyles and morphological characteristics [1,2,3]. Despite these similarities, the evolutionary relationships and placement of *Ylistrum* within the family Pectinidae are not conclusively determined. Molecular phylogenetic studies have indicated that the *Ylistrum* species is genetically distinct from its close relatives in the genus *Amusium* studied by Alejandrino et al. [4], Mynhardt et al. [1], and Sherratt et al. [5] who support this genetic separation. However, the precise phylogenetic placement of *Ylistrum* within the family remains a topic of debate. Serb [6] proposed that the *Ylistrum* might belong to the tribe Decatopectinini, but this suggestion has not been strongly supported by available evidence due to insufficient molecular data [1].

*Ylistrum* species are known for their long-distance swimming or gliding abilities, and their distinctive features include colorful valves and radiating interior ribs on both valves [1,3,7]. Two extant species within the *Ylistrum* genus are found across the globe: *Y*. *japonicum* (Gmelin, 1971) was originally discovered in Japan [8], and *Ylistrum balloti* (Bernardi, 1861), which is predominantly encountered in Australia’s western, eastern, and southern regions [3,9], and New Caledonia [10,11]. There is also a known fossil species from Morgan Limestone [12], *Ylistrum morganense* (Beu and Darragh, 2001). Species *Y*. *balloti* is pivotal to the commercial trawl fisheries in Australia, warranting extensive research. In contrast, studies on *Y*. *japonicum* are scarcer, with most research originating from Japan and South Korea. Okada [13] has delved into the ecology and morphology aspects of the species, and Kanmizutaru and Anraku [14] have investigated the effects of MgCl_2_ injection into the adductor muscle for shell opening. Kanmizutaru et al. [15] have evaluated the light perception capabilities of the pallial eyes through electroretinogram tests. In South Korea, studies have illuminated its reproductive cycle [16], the development of its gonads, the age at first sexual maturity, and the sex ratio [17], as well as the correlation between age and growth [18]. In China, there is limited research available, focusing primarily on ecology and the possibilities of artificial breeding [19,20].

The molecular dataset of *Y*. *japonicum* remains insufficient [1]. Before this study, there were nine nucleotides’ data about *Y*. *japonicum* in the NCBI GenBank database, including ribosomal RNA (12S rRNA, 16S rRNA, 18S rRNA, and 28S rRNA), partial sequences of protein-coding genes (cox1, nad1), and histone H3 gene. The taxonomic placement of *Y*. *japonicum* remains unsolved due to a lack of molecular data. A recent taxonomic investigation by Dijkstra and Beu [3] has provisionally maintained *Ylistrum* within the Amusiini tribe, awaiting a conclusive molecular phylogenetic analysis of the Pectinidae family.

In this study, the complete mitochondrial genome of *Y*. *japonicum* was analyzed, supplemented by the molecular data, and based on this result, phylogenetic studies were conducted to clarify the phylogenetic status of *Y*. *japonicum* within the family Pectinidae.

## 2. Results

### 2.1. Mitochondrial Genome Composition

**Figure 1:**
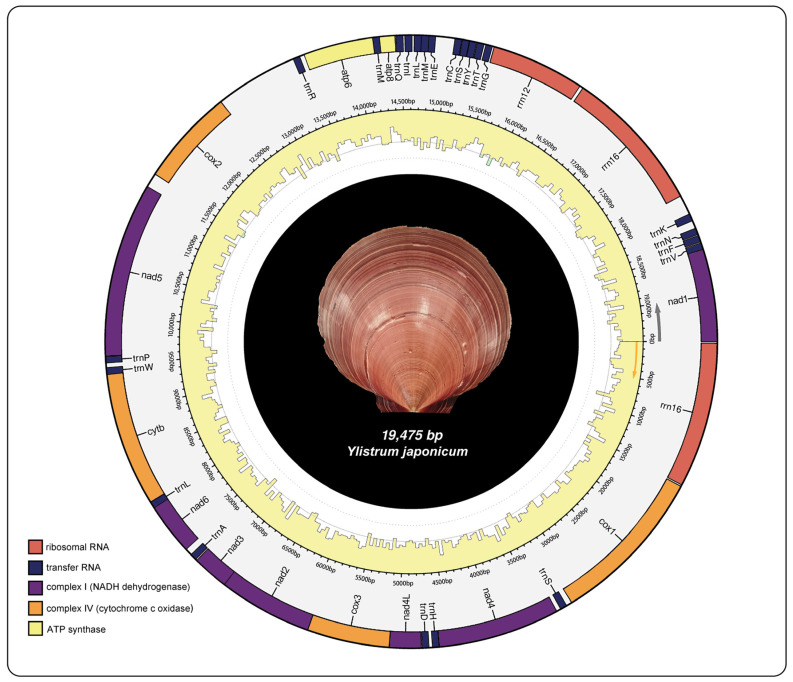
Mitochondrial genome map of *Ylistrum japonicum*.

The mitochondrial genome structure of *Y*. *japonicum* is depicted in Figure 1 and further described in Table 1. The complete mitochondrial genome sequence has been submitted to the NCBI GenBank database (Accession number: PP571649). The genome is characterized by a typical circular, closed, double-stranded structure with a total length of 19,475 bp. It encodes 39 genes, which include 13 protein-coding genes (PCGs), three ribosomal RNAs, and 23 transfer RNAs. The nucleotide composition is as follows: (A) constitutes 21.9%, thymine (T) 36%, guanine (G) 29%, and cytosine (C) 13.1%. The mitochondrial genome exhibits an A + T content of 57.9% and a G + C content of 42.1%.

### 2.2. Protein-Coding Genes

There are a total of 13 protein-coding genes; 12 out of the 13 protein-coding genes (PCGs) of *Y*. *japonicum* are commonly found in the majority of most pectinid species [21,22]. All genes are transcribed from the same strand and code in the same direction. Notably, gene *atp8*, which is typically absent in most mitogenomes of most bivalves [23,24], is present in *Y*. *japonicaum*. The total length of the PCGs is 11884 bp, comprising approximately 61% of the complete genome. Among the genes, only four (*atp6*, *cox1*, *cox2*, *nad4L*) utilize the standard start codon ATG. The remaining nine have alternative start codons, six genes (*atp8*, *cox3*, *nad1*, *nad2*, *nad4*, *nad6*) have GTG, two TTG (*cytb*, *nad5*), and *nad3* has ATC. Seven genes have the TAG stop codon, five have TAA, and cytb is terminated by T.

### 2.3. rRNA and tRNA Genes

The *rrnS* (*rrn12*) gene spans 957 bp (from position 15459 to 16415), while the *rrnL* (*rrn16*) gene has two copies with lengths of 1492 bp and 1486 bp (18-1509, 16453-17939, respectively). The mitochondrial genome of *Y*. *japonicum* contains 23 tRNA genes ranging in length from 65 to 72 nucleotides. Three tRNA genes are present in two copies; all three tRNA genes are found with distinct anticodons. Two *trnS* (*rRNA-Ser*) have UCU or UGA, two *trnL* (*rRNA-Leu*) have UAA or UAG, and two *trnM* (*rRNA-Met*) have UAU or CAU. The occurrence of multiple *trnM* genes in the mitochondrial genomes of bivalves is common [21], and in the majority of mitochondrial genomes of animals, the occurrence of two copies of *trnS* is frequently noted, as reported by Malkócs et al. [25].

### 2.4. Control Region

The mitogenome of *Y. japonicum*, akin to the majority of bivalve species, contains a substantial complement of unassigned nucleotides. In contrast to other scallop species, *Y. japonicum* exhibits a great number of control regions despite having a shorter major sequence length. Specifically, the mitochondrial genome of *Y. japonicum* contains a total of 38 control regions, with 5764 bp of intergenic control region nucleotides in which the longest continuous sequence (855bp) is between the *cox2* and *trnR* genes, accounting for approximately 14.83% of all unassigned nucleotides.

### 2.5. Gene Order

**Figure 2:**
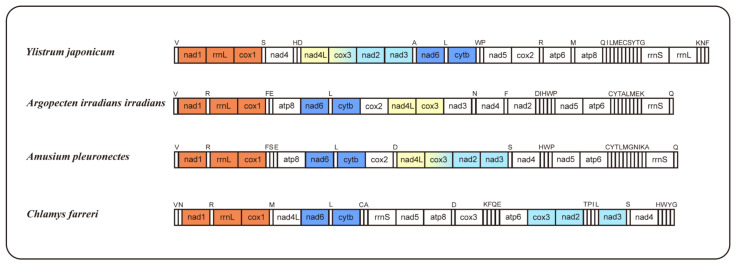
Gene orders of *Ylistrum japonicum*, *Argopecten irradians irradians*, *Amusium pleuronectes*, and *Chlamys farreri*, with newly annotated atp8 genes. The same color indicates identical gene junctions (excluding the tRNA genes).

The occurrence of mitogenome rearrangements is prevalent among mollusks [26]; the arrangement observed in the mitogenome of *Y. japonicum* is also a novel configuration for the family Pectinidae, with no matching gene junctions found in other Pectinidae species (Figure 2). Species with higher gene order similarity were selected for comparison and newly annotated atp8 genes, according to Malkócs et al. [25]. Due to the lack of annotation of rRNA sequence, *Mizuhopecten yessoensis* (FJ595959) is excluded from the gene order analysis, and based on the high similarity of gene order between three *Argopecten* species [27], only one species was selected as a representative.

Comparing gene arrangements of four selected species, a conserved gene cluster, “nad6-trnL-cytb,” was identified as common to all. When excluding the tRNA genes, an additional shared gene cluster, “nad1-rrnL-cox1,” was observed among the four species. Gene cluster “nad4L-cox3” is present in *Y*. *japonicum*, *Argopecten irradians irradians*, and *Amusium pleuronectes*, while gene cluster “cox3-nad2-nad3” is shared by *Y*. *japonicum*, *A*. *pleuronectes*, and *Chlamys farreri*. The “nad5-atp6” cluster in *Y*. *japonicum* is split by the insertion of cox2, distinguishing it from the other two *Pectininae* species. The “nad5-atp6-rrnS” cluster, excluding variable tRNA genes, is also shared between *A*. *irradians irradians* and *A*. *pleuronectes*, indicating a close evolutionary relationship between the two species.

### 2.6. Gene Collinearity

**Figure 3:**
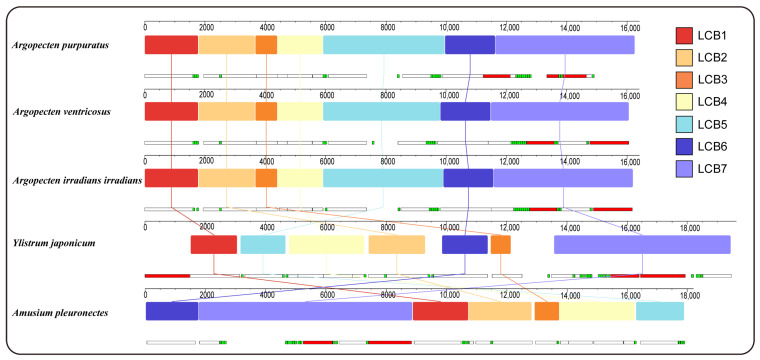
Gene collinearity analysis of 5 Pectininae species. The level of similarity at each position is shown in the blocks. The white, red, and green boxes represent protein-coding, rRNA, and tRNA genes.

Gene collinearity analysis using the progressiveMauve algorithm in Mauve has identified seven locally collinear blocks (LCBs) across the complete mitochondrial genome of five Pectininae species (Figure 3). These LCBs are conserved across all mitogenomes analyzed, although variations in the sequence order are evident among the different species. The arrangement of LCBs demonstrated a high degree of similarity among the three *Argopecten* species, indicating their close evolutionary relationship. In contrast, *Y*. *japonicum* exhibited a significantly dissimilar LBCs arrangement compared to three *Argopecten* species and *A*. *pleuronectes*.

### 2.7. Phylogenetic Analysis

**Figure 4:**
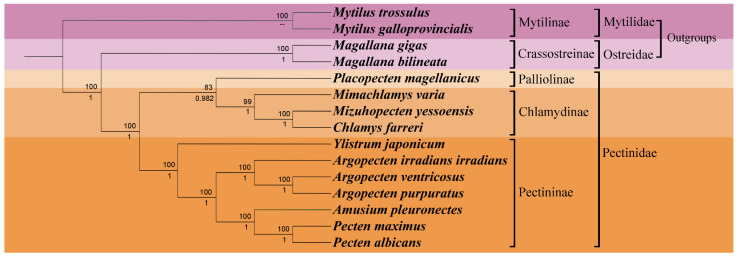
Phylogenetic tree derived from Maximum likelihood (ML) and Bayesian inference (BI) based on the sequences of mitochondrial protein-coding genes (PCGs). Numbers above the branches indicate bootstrap support; numbers below branches are Bayesian posterior probability. A dash indicates no support for that node.

To delve deeper into the phylogenetic position of *Y*. *japonicum* and the taxonomic status within the family Pectinidae, a phylogenetic tree (Figure 4) was constructed based on complete or nearly complete mitochondrial genome data of various Pectinidae species and outgroup taxa. The results of the phylogenetic analysis were found to be comparable with previous studies by Smedley et al. [28], Yao et al. [29], and Malkócs et al. [25] and largely adhered to the taxonomic framework established by Waller [30,31]. Phylogenies based on two methods (Maximum Likelihood and Bayesian inference) of the concatenated protein sequences show almost complete agreement, with high bootstrap values or posterior probabilities supporting all nodes. The systematic arrangement, as proposed by Waller [31], subdivides the family Pectindae into four subfamilies: Pectininae, Chlamydinae, Pallioline, and Camptonectinae. The outgroup Mytilinae and Crassotreinae are found to be consistent with the phylogenetic position proposed by Xu et al. [21], where the clade Mytilinae forms a sister group with the clade Osteridae + Pectinidae.

Our study has validated the earlier proposed hypothesis regarding the monophyly of Pectinidae, as initially concluded by Waller [32]. Nevertheless, the phylogenetic analysis in our study was unable to include any representatives from the Camptonectinae subfamily due to the lack of complete mitochondrial genome sequences available for this group. The Pectinidae species were effectively categorized into three subfamilies: Palliolinae, Chlamydinae, and Pectininae. *Placopecten magellanicus*, serving as the representative of the Palliolinae, was positioned at the basal position of the branch Palliolinae + Chlamydinae. The clade Palliolinae + Chlamydinae was well supported as the sister group to the Pectininae clade [33,34]. Within the subfamily Chlamydinae, *M*. *yessonesis* and *C. farreri* were found to be the most related, forming a sister taxon relationship with *Mimachlamys*, which aligns with the findings of Xu et al. [21]. *Ylistrum* was identified as a basal lineage to the clade Pectininae, separated from its previously assigned tribe Amusiini, a conclusion that is in agreement with the work of Alejandrino et al. [4], Sherratt et al. [5], and Serb [6]. *Argopecten* species were clustered on the same branch, forming a sister group with the clade composed of *Amusium* + *Pecten*. The close relationship between *A*. *pleuronectes* and the clade of *Pecten maximus* + *Pecten albicans* was also consistent with the research conducted by Barucca et al. [35], Alejandrino et al. [4], and Feng et al. [34].

**Figure 5:**
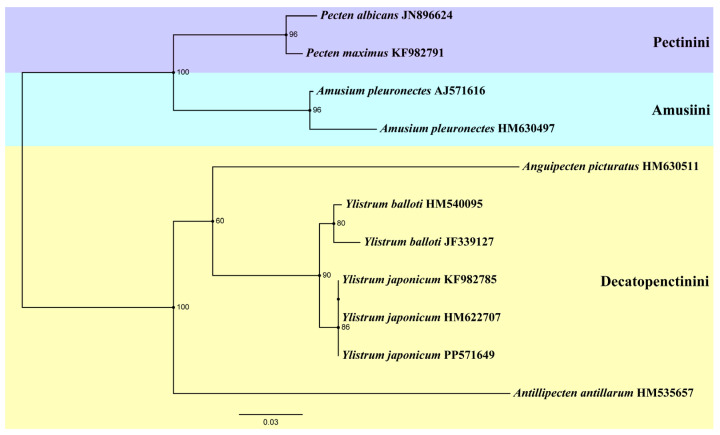
Phylogenetic tree of genus *Ylistrum* and some species from three tribes of Pectininae inferred by Maximum likelihood (ML) of 16S rRNA sequences. Numbers indicate bootstrap support.

Another ML tree (Figure 5) was constructed based on 16S rRNA sequences, incorporating three specimens of *Y*. *japonicum* from China (PP571649) and Japan (HM622707, KF982785) [4,36]. The genus *Amusium* and *Pecten* form a sister group again. *Antillipecten antillarum* is positioned as a basal lineage, forming a sister group with the clade consisting of *Anguipecten* + *Ylistrum*. The two *Ylistrum* species form a sister clade and are well separated from *Amusium*. This analysis aligns with the findings of Mynhardt et al. [1]. The Japanese specimens HM622707 and KF982785 are grouped on the same branch, indicating a shared ancestry with the Chinese individuals. All *Y*. *japonicum* finally converged into the same branch, showing the close genetic distance between its individuals.

### 2.8. Systematic Descriptions

Order Pectinida Gray, 1854

Superfamily Pectinoidea Rafnesque, 1815

Family Pectinidae Rafnesque, 1815

Subfamily Pectininae Rafnesque, 1815

Tribe Decatopectinini Waller, 1986

Genus *Ylistrum* Mynhardt and Alejandrino, 2014

*Ylistrum japonicum* (Gmelin, 1791)

*Type locality*: Japan.

*Distribution*: Japan (South of central Honshu Island), Korea (Jeju Island), China (Taiwan Province, Guangdong Province, Guangxi Province, and Hainan Province).

Morphological description: Shell large, round, smooth, and glossy. The left valve is dark red to reddish-brown, covered with concentrically arranged dark brown fine lines and spots. The color is slightly lighter at the umbo, with small light-colored spots. The right valve is slightly flat, pale yellow to light tan, white near the umbo, with concentrically arranged brown spots on the surface. Two small auricles are slightly different in size; the color of the auricles on the left valve is darker. The inner surface of the shell is white, with the left valve having a yellow to light brown edge; sometimes, the inner edge of the left valve is pale brown. Interior radial ribbing is on both valves; the specimen was collected from Hailing Island with 33–43 ribs on the left valve and 42–49 ribs on the right valve.

*Remarks*: In the original description, the species group from China was recorded as a subspecies *Amusium japonicum taiwanicum* Habe, 1992 [37], and now it has a synonymized name of *Y. japonicum*. Unlike individuals from Japan, the color of specimens from China are not bright, and the concentrically arranged brown spots are present on the right valve; these are the morphological differences between the individuals from the above two producing areas. Despite their morphological differences, their molecular biological evidence indicates that they are the same species. The counting of the internal ribs by different authors is not always the same (e.g., Zhang et al. [38], Wang [7], Mynhardt et al. [1]). In addition, counts had completely overlapping ranges, and they could not be used to differentiate between the two *Ylistrum* species [1]. Overall, the most significant difference between Y. japonicum and *Y. balloti* is the color of their auricles on the right valve and the spots in a concentric pattern on both two valves. To the former species, the auricles on the right valve are generally darker, and spots always appear along with their valve repair marks.

When mixing all the scallops obtained from trawl nets, 5 out of 52 scallops are *Y*. *japonicum* in one trawl, with sandy bottom sediment, and others are *A*. *pleuronectes*. The average shell length of *Y*. *japonicum* in the population is 84.27 ± 10.63 mm (*n* = 15), the average shell height is 82.97 ± 9.48 mm, and the average shell width is 16.70 ± 2.23 mm.

## 3. Discussion

The family Pectinidae, as a clade within Bivalvia, exhibits a significant range of morphological and behavioral variations, rendering it of great importance in ecology, evolution, and commercial activities. Nevertheless, the taxonomy of Pectinidae has long been a source of debate within the scientific community.

Waller’s [30] hypothesis for the classification and evolution of Pectinidae is based on morphology, particularly focusing on pre-radial stage shell microsculpture and incorporating fossil data and geological evidence. In 2006, Waller updated his Pectinidae phylogenetic hypothesis in conjunction with previous molecular genetic studies about the phylogenies analysis of the Pectinidae, and a stable classification method was successfully established. Much of the contemporary taxonomic research on Pectinidae is grounded in the taxonomy framework developed by Waller, as evidenced by the work of Serb [6] and Smedley et al. [28]. With the continuous development of molecular technology and the expansion of fossil evidence, phylogenetic studies of Pectinidae have been further improved; however, controversies over the correct classification of this family remain.

In molecular phylogenetic studies, discrepancies in research can arise from numerous sources, including the selection of a single genetic sequence [39] or the combination of multiple sequences [40,41]. Moreover, the precision and constraints of methods used to construct phylogenetic trees [6,42,43], the quantity and diversity of species examined, the choice of outgroups, and the handling of sequences all play a role in shaping the final results. A prime example of such inconsistencies is seen in the phylogenetic positioning of the Palliolinae, a monophyletic subgroup within the family Pectinidae. Various studies have placed the Palliolinae in different clades, either with the Pectininae or the Chlamyinae, illustrating the persistent challenges in achieving phylogenetic resolution within this family (e.g., Alejandrino et al. [4], Sherratt et al. [5], Xu et al. [21], Lin et al. [22], Malkócs et al. [25], Li et al. [27], Smedley et al. [28], Saavedra and Peña [33], Feng et al. [34], and Malkowsky and Klussmann-Kolb [40]). In contrast, Waller’s hypothesis proposed that the Palliolinae are sisters to the Pectininae [31]. On the mito-phylogenomics level, differences in sequence selection and methodologies can lead to varying results, as evidenced by Lin et al. [22], Malkócs et al. [25], and Li et al. [27]. The divergence time estimation analysis conducted by Lin et al. [22] based on concatenated mitochondrial protein-coding gene sequences produced a phylogenetic structure akin to our own findings, hinting that variations in outcomes might be linked to the selection of samples. In conclusion, the discrepancies observed in phylogenetic studies can be ascribed to the diversity in sequence choices, analytical techniques, and the species included in the sampling.

Although the robustness of a phylogenetic tree can be affected by a variety of factors, it is noteworthy that *Ylistrum* and *Amusium* have consistently been distinguished in previous molecular phylogenetic studies. The placement of *Ylistrum* within the subfamily Pectininae is well-supported, as evidenced by the study of Matsumoto and Hayami [39] and subsequent research [4,28]. Nevertheless, due to the significant morphological and distributional similarities between *Y*. *japonicum* and *A*. *pleuronectes*, *Ylistrum* has historically been grouped with the Amusiini, even though molecular genetic studies have consistently pointed to its distinctiveness from *Amusium*. Our phylogenetic study based on the complete mitochondrial genome indicates that *Ylistrum* has an ancient origin, but its precise placement within the subfamily Pectininae remains ambiguous due to insufficient sample data. Alejandrino et al. [4] analyzed the phylogeny of 81 extant taxa from the Pectinidae based on the nuclear Histone H3, 12S rRNA, 16S rRNA data, and 28S rRNA data, and the result shows that *Ylistrum* was placed among the species of the tribe Decatopectinini, and *Aumsium* were nested in a different clade (Pectinini). Subsequently, Smedley et al. [28] expanded the dataset to 62 Pectinidae species on the basis of Alejandrino et al. [4] into a new phylogenetic analysis, and clade *Ylistrum* was once again placed in the Decatopectinini. Interestingly, it forms a sister clade with two *Annachlamys* species belonging to the tribe Pectinini, but the phylogenetic location of *Annachlamys* is still debated [3]. Mynhardt et al. [1] focused on the phylogenetic analysis of *Aumsium* and *Ylistrum*, restored their respective monophyletic clades, and described *Ylistrum* as a new genus. In this study, *Ylistrum* also forms a sister group with a Decatopectinini species (*A*. *antillarum*). Our phylogenetic analysis based on 16S rRNA indicates the same result as that of former studies: two *Ylistrum* taxa are still nested in the tribe Decatopectinini. In light of these findings and previous studies, we adhere to Serb’s classification [6], positioning *Ylistrum* within the Decatopectinini tribe.

The complete mitochondrial genome sequence data for *Ylistrum* remain inadequate. The available data for *Y*. *balloti* (accession number ON041136) may be based on an erroneous identification. NCBI-BLAST analysis of this sequence, with an alignment length exceeding 1200 nucleotides, reveals a similarity greater than 98.99% with *Y*. *japonicum* but less than 94.10% with *Y*. *balloti*. This indicates that ON041136 is likely a misidentified *Y*. *japonicum* rather than *Y*. *balloti*. The researcher who submitted this sample has not published their findings, and morphological verification is not possible. Additionally, the collection location of ON041136 is Beihai, Guangxi, China, which is problematic since there are no documented distributions of *Y*. *balloti* within China. Generally speaking, the right valve of *Y*. *balloti* is white or pale brown, with concentric, irregularly sized violet–brown spots [1], rather than a white, unspotted right valve, so the mention of *Y*. *balloti* in the Chinese Zoology book [7] is considered a misidentification, highlighting the need for a critical revision of this information.

## 4. Materials and Methods

### 4.1. Sample Collection

**Figure 6:**
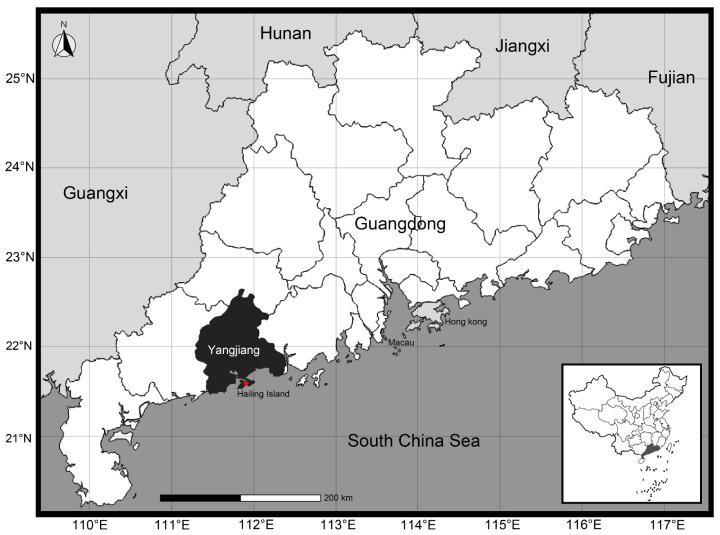
Sampling location of *Ylistrum japonicum* (modified from d-maps: https://d-maps.com, accessed on 10 May 2024).

A total of 50 specimens were collected from 2022 to 2023 from Hailing Island, Yangjiang City, Guangdong Province, China (21.61 N, 111.93 E) (Figure 6). Morphometric measurements were performed using an electronic vernier caliper (0.1 mm), and body measurement traits (shell length, shell height, shell width, shell weight, etc.) were recorded for further investigation. All collected samples were intended for commercial purposes, and there were no concerns regarding animal ethics. The morphological characteristics of these specimens were categorized and compared in accordance with Zhang et al. [38] and Zhang [44].

### 4.2. DNA Extraction, Library Preparation, and Next Generation Sequence

One specimen had its adductor muscles extracted (5 g) for DNA extraction; gDNA was extracted by the MagPure Bacterial DNA Kit (Magen, Guangzhou, China) following pre-grinding in liquid nitrogen. The Qubit dsDNA HS assay kit (Sangon, Shanghai, China) was used to test the concentration and 1% agarose gel electrophoresis to confirm integrity. The library preparation and next-generation sequence were finished by Sangon Biotech (Shanghai) Co., Ltd. First, 500 ng quantified DNA was randomly fragmented by Covaris (Woburn, MA, USA). Next, Hieff NGS^®®^MaxUp II DNA Library Prep Kit for Illumina^®®^ (YEASEN, Shanghai, China) was used for the next steps. Briefly, Endprep enzyme was added to repair the end and 3’ end A tail ligation. Then, the adaptor was ligated by an enhancer and Fast T4 DNA ligase. Index primer was added by PCR, and the amplified product, about 400 bp, was selected using DNA selection beads. The library concentration and size were confirmed by Qubit 4.0 (Thermo, Waltham, MA, USA) and 2% agarose gel electrophoresis, respectively.

Then, the libraries were pooled and loaded on the Novaseq 6000 (Illumina. San Diego, CA, USA)/DNBseq-T7 (BGI, Shenzhen, China) sequencer using the 2×150 bp paired-end sequence kit according to the manufacturer´s instructions.

### 4.3. Sequence Assembly and Annotation

Raw bases yielded at least 6 GB and were used for downstream analysis. First, all of the raw reads were trimmed by Fastqc v0.11.2 [45]. The software SPAdes v3.15 [46] was used to assemble the raw sequence reads into contigs. tBLASTn v2.6.0 and GeneWise were used to obtain the CDS gene boundary by reverse alignment with the near-source reference database; the tRNA sequence annotation was obtained by MiTFi, Rfam used cmsearch alignment to identify non-coding rRNA, and the final summary was put into complete annotation results. The circular gene maps of the species *Y*. *japonicum* were drawn by Circos v0.69.

### 4.4. Gene Collinearity

Gene collinearity among complete mitochondrial sequences of five Pectininae species was explored to assess their phylogenetic relationship with *Y*. *japonicum*, using the progressiveMauve algorithm and default parameters (including default seed weight, determine locally collinear blocks and full alignment) in the Mauve v2.4.0 [47].

### 4.5. Phylogenetic Analysis with Mitochondrial Genome

Two phylogenetic analyses were conducted based on the complete mitochondrial sequences of *Y*. *japonicum* in this study. Following the methodologies established in previous studies by Malkowsky and Klussmann-Kolb [39], Xu et al. [21], and Malkócs et al. [25], a total of 15 mitochondrial sequences of Pectinidae species and outgroup taxa were selected to construct phylogenetic trees. This selection included 11 Pectinidae species across three subfamilies: Pectininae, Palliolinae, and Chlamydinae. The available mitochondrial genome sequences were obtained from GeneBank, incorporating 13 complete mitochondrial genomes and 2 incomplete sequences (KP900974, KP900975), each over 16,000 base pairs in length. Two Ostreidae species, *Magallana bilineata* and *Magallana gigas,* and two Mytilidae species, *Mytilus galloprovincialis* and *Mytilus trossulus*, were used as outgroups.

PhyloSuite v1.2.3 [48] was utilized to extract the protein-coding genes (PCGs) from each sequence. All sequences were aligned in batches with MAFFT v7.505 [49]. The alignments were refined using the codon-aware program MACSE v2.06 [50], which preserves the reading frame and allows the incorporation of sequencing errors or sequences with frameshifts. Ambiguously aligned fragments of the alignments were removed in batches using Gblocks 0.91b [51]. ModelFinder v2.2.0 [52] was used to select the best-fit partition model. The phylogenetic tree was subsequently constructed using both the Maximum likelihood (ML) method in IQ-TREE v2.2.0 [53,54] and Bayesian inference (BI) in MrBayes v3.2.7a [55]. Branch support was determined with 5000 bootstrap iterations for the best-scoring ML tree. Markov Chain Monte Carlo (MCMC) analyses were run for 1,000,000 generations (sampling every 1000 generations), in which an initial 50% of sampled data were discarded as burn-in. The result was beautified with FigTree v1.4.4.

To explore in more detail the monophyletic development of *Y*. *japonicum* and its taxonomic position in Pectininae, another ML tree was constructed using 16S rRNA by PhyloSuite v1.2.3 [48]; three *Y*. *japonicum* specimens from different regions (China and Japan) and the other 8 specimens of subfamily Pectininae were selected. All Sequences were aligned in batches with MAFFT v7.505 [49] and pruned by Gblocks 0.91b [51]. Branch support was determined with 5000 bootstrap iterations for the best-scoring ML tree. A list of specimens included in molecular studies is shown in Table 2.

## Figures and Tables

**Table 1:** Structural features of the mitochondrial genome of *Ylistrum japonicum*.

Gene	Sequence Location	Size (bp)	Start Codon	Stop Codon	Intergenic Nucleotide (bp)
*rrnL*	18–1509	1492			17
*cox1*	1527–3158	1632	ATG	TAA	17
*trnS*	3219–3286	68			60
*nad4*	3334–4581	1248	GTG	TAG	47
*trnH*	4588–4652	65			6
*trnD*	4690–4757	68			37
*nad4L*	4769–5089	321	ATG	TAA	11
*cox3*	5094–5939	846	GTG	TAG	4
*nad2*	5942–6904	963	GTG	TAA	2
*nad3*	6904–7275	372	ATC	TAA	−1
*trnA*	7291–7356	66			15
*nad6*	7431–7958	528	GTG	TAA	74
*trnL*	7960–8024	65			1
*cytb*	8028–9402	1375	TTG	T	3
*trnW*	9403–9471	69			0
*trnP*	9522–9586	65			50
*nad5*	9594–11,381	1788	TTG	TAG	7
*cox2*	11,532–12,524	993	ATG	TAG	150
*trnR*	13,380–13,447	68			855
*atp6*	13,498–14,211	714	ATG	TAG	50
*trnM*	14,216–14,280	65			4
*atp8*	14,284–14,439	156	GTG	TAG	3
*trnQ*	14,451–14,521	71			11
*trnI*	14,542–14,612	71			20
*trnL*	14,638–14,706	69			25
*trnM*	14,713–14,784	72			6
*trnE*	14,788–14,853	66			3
*trnC*	15,053–15,118	66			199
*trnS*	15,129–15,195	67			10
*trnY*	15,205–15,271	68			9
*trnT*	15,283–15,350	68			11
*trnG*	15,372–15,437	66			21
*rrnS*	15,459–16,415	957			21
*rrnL*	16,453–17,939	1486			37
*trnK*	18,145–18,216	72			205
*trnN*	18,301–18,367	67			84
*trnF*	18,381–18,445	65			13
*trnV*	18,459–18,525	67			13
*nad1*	18,528–19,475	948	GTG	TAG	2

**Table 2:** List of specimens included in the molecular studies.

Subfamily	Tribe in This Research	Previous Tribe	Species	Genbank Accession Numbers	Genetic Compartments
Ingroup					
	Aequipectinini	Aequipectinini	*Argopecten irradians irradians*	DQ665851	mitogenome
	Aequipectinini	Aequipectinini	*Argopecten purpuratus*	KT161260	mitogenome
	Aequipectinini	Aequipectinini	*Argopecten ventricosus*	KT161261	mitogenome
Pectininae	Amusiini	Amusiini	*Amusium pleuronectes*	MT419374	mitogenome
	Amusiini	Amusiini	*Amusium pleuronectes*	AJ571616	16S rRNA
	Amusiini	Amusiini	*Amusium pleuronectes*	HM630497	16S rRNA
	Decatopectinini	Amusiini	*Ylistrum japonicum*	PP571649	mitogenome
	Decatopectinini	Amusiini	*Ylistrum japonicum*	KF982785	16S rRNA
	Decatopectinini	Amusiini	*Ylistrum japonicum*	HM622707	16S rRNA
	Decatopectinini	Amusiini	*Ylistrum balloti*	HM540095	16S rRNA
	Decatopectinini	Amusiini	*Ylistrum balloti*	JF339127	16S rRNA
	Decatopectinini	Decatopectinini	*Anguipecten picturatus*	HM630511	16S rRNA
	Decatopectinini	Decatopectinini	*Antillipecten antillarum*	HMS35657	16S rRNA
	Pectinini	Pectinini	*Pecten maximus*	KP900975	mitogenome
	Pectinini	Pectinini	*Pecten maximus*	KF982791	16S rRNA
	Pectinini	Pectinini	*Pecten albicans*	KP900974	mitogenome
	Pectinini	Pectinini	*Pecten maximus*	JN896624	16S rRNA
Palliolinae	Palliolini	Palliolini	*Placopecten magellanicus*	DQ088274	mitogenome
	Chlamydini	Chlamydini	*Chlamy farreri*	EF473269	mitogenome
Chlamydinae	Fortipectinini	Fortipectinini	*Mizuhopecten yessoensis*	FJ595959	mitogenome
	Mimachlamydini	Mimachlamydini	*Mimachlamys varia*	MZ520326	mitogenome
Outgroup					
Crassostreinae			*Magallana bilineata*	MT985154	mitogenome
			*Magallana gigas*	MZ497416	mitogenome
Mytilinae			*Mytilus galloprovincialis*	DQ399833	mitogenome
			*Mytilus trossulus*	AY823625	mitogenome

## Data Availability

Data are contained within the article.
